# Noninvasive Assessment of Liver Fibrosis with ElastPQ in Patients with Chronic Viral Hepatitis: Comparison Using Histopathological Findings

**DOI:** 10.3390/diagnostics12030706

**Published:** 2022-03-14

**Authors:** Dongmin Choo, Kyung Sook Shin, Ji Hye Min, Sun-kyoung You, Kyung-Hee Kim, Jeong Eun Lee

**Affiliations:** 1 Sok Medical Clinic, 586 Gyeryong-ro, Seo-gu, Daejeon 35300, Korea; choo3421@cnuh.co.kr; 2 Department of Radiology, Chungnam National University Hospital, College of Medicine, Chungnam National University, 282 Munhwa-ro, Jung-gu, Daejeon 35015, Korea; shinks@cnu.ac.kr (K.S.S.); sunkyou@hanmail.net (S.-k.Y.); 3 Department of Radiology and Center for Imaging Science, Samsung Medical Center, School of Medicine, Sungkyunkwan University, 81 Irwon-ro, Gangnam-gu, Seoul 06351, Korea; minjh1123@gmail.com; 4 Department of Pathology, Chungnam National University Sejong Hospital, College of Medicine, Chungnam National University, 282 Munhwa-ro, Jung-gu, Daejeon 35015, Korea; phone330@cnu.ac.kr

**Keywords:** elastography point quantification (ElastPQ), liver stiffness measurement, liver fibrosis staging, chronic viral hepatitis, shear wave elastography

## Abstract

Chronic viral hepatitis is a major cause of chronic liver disease leading to liver fibrosis. This study aimed to assess the diagnostic performance of elastography point quantification (ElastPQ), transient elastography (TE), and aspartate aminotransferase-to-platelet count ratio index (APRI) for the staging of liver fibrosis in patients with chronic viral hepatitis using histopathological findings as a reference standard. For 122 patients with chronic viral hepatitis, diagnostic performance was evaluated using area under the receiver operating characteristic curve (AUROC) analysis and correlations were determined using Spearman’s correlation coefficient. The AUROC of ElastPQ for the diagnosis of the fibrosis stage ≥ F2 was 0.917 with a cut-off value of 3.935. There was a significant positive correlation between the different stages of histologic liver fibrosis and stiffness values obtained using ElastPQ, TE, and APRI (ρ = 0.556, ρ = 0.657, ρ = 0.375, respectively; *p* < 0.001). ElastPQ showed a higher diagnostic accuracy than APRI, resembling that of TE; AUROC values of ElastPQ, TE, and APRI were 0.917, 0.964, and 0.896, respectively, for a fibrosis stage ≥ F2. ElastPQ is a promising noninvasive technique with a diagnostic accuracy comparable with that of TE for the evaluation of liver fibrosis in patients with chronic viral hepatitis.

## 1. Introduction

Chronic viral hepatitis caused by hepatitis B virus (HBV) and hepatitis C virus (HCV) is one of the most common causes of chronic liver disease that can lead to liver cirrhosis and associated sequelae [1]. The management of chronic liver disease and its prognosis mainly depends on the extent and severity of liver fibrosis [2]. Although liver biopsy has been considered the gold standard for the assessment of liver fibrosis [3], it has several limitations, including many procedure-related complications, sampling errors, intra- and inter-observer variability, and over- and under-staging of fibrosis [4,5]. Therefore, most studies have focused on the evaluation of noninvasive methods for the staging of liver fibrosis. More recently, non-invasive methods including serologic fibrosis marker tests such as the aspartate aminotransferase (AST)-to-platelet count (PLT) ratio index (APRI) and various elastography techniques including magnetic resonance elastography and ultrasound (US) elastography have increasingly been used for the assessment of liver fibrosis [6,7,8,9,10].

Most US-based elastography techniques including transient elastography (TE), acoustic radiation force impulse (ARFI) imaging, elastography point quantification (ElastPQ), and two-dimensional shear wave elastography use shear waves for absolute soft-tissue stiffness quantification. Among various US-based elastography techniques, TE is the most widely used and is an important method for the diagnosis of liver fibrosis; its usefulness has been confirmed in several meta-analyses [11,12,13]. However, TE has certain limitations regarding liver-stiffness measurements in patients with narrow intercostal spaces, ascites, or obesity [14].

Point shear wave elastography, such as ElastPQ and ARFI imaging using shear waves, are reliable tools for evaluating liver fibrosis [15,16]. Unlike TE, ElastPQ and ARFI can be implemented into conventional US devices and can be used to assess liver fibrosis in patients with chronic viral hepatitis during an abdominal US examination [17]. While the diagnostic performance of ARFI has been demonstrated to be comparable to that of TE in meta-analyses, studies on ElastPQ are limited [9,18,19]. Recently, there have been several studies comparing the diagnostic performance of ElastPQ and TE indicating the good diagnostic performance of ElastPQ for noninvasive liver-fibrosis staging in patients with chronic liver disease [20,21,22,23]. However, some of these studies did not use liver biopsy as a reference standard [17,23,24]. Indeed, the diagnostic performance of elastography technology is influenced by the underlying cause of liver disease [18,25]. While previous studies have compared the performance of ElastPQ and other noninvasive tools for chronic liver disease patients, using TE as a reference standard, this study solely focused on chronic viral hepatitis patients and used histopathologic findings as a standard reference. Our aim was to compare the diagnostic performance of ElastPQ, TE, and serologic fibrosis markers in chronic viral hepatitis patients using histopathologic findings as the reference standard.

## 2. Materials and Methods

### 2.1. Patients and Study Design

**Figure 1:**
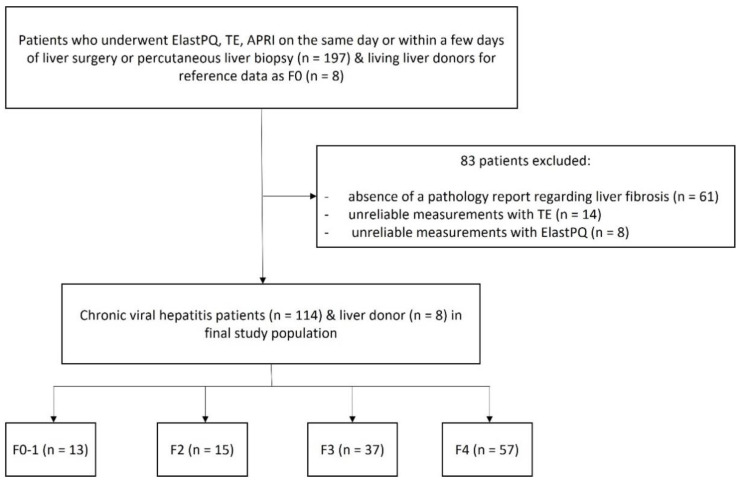
Flow chart of the study population.

This retrospective study was approved by the respective institutional review board and the requirement for informed consent was waived considering the minimal risk involved in the study. Patient information was obtained using electronic medical records. We reviewed 197 patients with virologically confirmed chronic viral hepatitis who had undergone liver-stiffness measurements using ElastPQ and TE on the same day or within a few days of liver biopsy or liver surgery at the Chungnam National University Hospital. Percutaneous liver biopsy or liver surgery was performed to obtain data on clinical indications such as the abnormal liver function test result or to resect focal hepatic malignancies. In addition, eight living liver donors who were examined using ElastPQ and TE were included in the patient group as the Metavir F0 (no fibrosis) pathology group [26]. Of the included patients, 83 were excluded owing to the absence of a liver fibrosis pathology report (*n* = 61), unreliable measurements with TE (*n* = 14), and unreliable measurements with ElastPQ (*n* = 8). The final study population comprised 114 patients with chronic viral hepatitis and 8 donors (Figure 1).

### 2.2. Point Shear Wave Elastography (ElastPQ)

ElastPQ was performed using a US system (iU-22, Philips Medical Systems, Bothell, WA, USA) by two physicians with 3 years of US elastography experience and 7 years of liver US experience who were blinded to the patients’ clinical information. The measurements were performed via the intercostal approach at the right lobe of the liver in a supine position [27]. Using B-mode imaging, the physician placed the measurement box (approximately 0.5 cm by 1.5 cm) at the region of interest (free of bile ducts or vessels) and then pressed the “update” button to measure the stiffness in real time. The liver stiffness value was expressed in kilopascals (kPa). In the case of non-shear wave motion over the threshold, the system displayed “0 kPa” owing to the calculation failure; <1 kPa was regarded as an invalid measurement. The median value of 10 valid measurements were obtained and recorded as the liver stiffness measurements on ElastPQ. If 10 valid measurements were not collected within 15 attempts, the evaluation was considered a failure. Based on a recent study, liver stiffness measurements with an interquartile range (IQR)/median value of ≤30% were considered reliable [28].

### 2.3. Transient Elastography (TE)

All patients underwent TE using a Fibroscan (Echosens, Paris, France) on the same day of the ElastPQ measurement or at least within two days. It was performed by two clinicians with over 4 years of experience in TE measurement. Liver stiffness measurements were performed through the intercostal spaces at the right lobe of the liver in a supine position. The tip of the transducer was placed on the skin overlying the right lobe of the liver. Following the appropriate placement of the measurement (a region at a depth of 25–65 mm), the physician pressed a button on the probe for data collection. An examination was considered reliable if 10 valid measurements had acquired a success rate of at least 60% and an IQR < 30%. The median was considered as the representative value.

### 2.4. Serological Test

All patients underwent serological and biochemical investigation. Serological tests were performed with overnight fasting on the same day as the liver biopsy or the day before surgery. The following serological markers were routinely evaluated: AST, alanine aminotransferase (ALT), alkaline phosphatase (ALP), albumin, r-glutamyltransferase (r-GT), and PLT. The APRI was used to assessed the indicators of the noninvasive serologic marker test for liver fibrosis which were calculated using the following formula: APRI = (AST/ASTULN × 100)/PLT, where ASTULN is defined as the upper limit of the normal AST value (40 IU/L) [29,30].

Chronic hepatitis B was diagnosed by a positive serology test for the serum hepatitis B surface antigen or HBV-DNA. Chronic hepatitis C was assessed based on the presence of antibodies against the HCV and HCV-RNA.

### 2.5. Histopathological Analysis

All specimens were obtained during surgery and by percutaneous liver biopsy. The specimens were fixed in 4% buffered formalin and subsequently embedded in paraffin blocks. Tissue sections (4 μm thick) were stained using hematoxylin and eosin and using Masson’s trichrome stain. All specimens were analyzed by a pathologist with 15 years of clinical experience. The pathologist was blinded to the patients’ medical records and the elastography results. The fibrosis grade evaluation was performed semi-quantitively according to the Metavir scoring system, as follows: F0 = no fibrosis, F1 = portal fibrosis without septa, F2 = portal fibrosis with a few septa, F3 = numerous septa without cirrhosis, and F4 = cirrhosis. Significant fibrosis was defined as F2 or greater (≥F2) [31].

### 2.6. Data and Statistical Analyses

All statistical analyses were performed using two different statistics software (SPSS, version 18, Chicago, IL, USA; MedCalc Software, version 18.11, Ostend, Belgium). All continuous variables are expressed as means ± standard deviations. Categorical variables are summarized as counts and percentages. The trend between the values of ElastPQ, TE, APRI, FIB-4, and pathologic fibrosis stage was analyzed using the Spearman’s correlation test. The median values of ElastPQ and TE were compared using the Mann–Whitney test. The diagnostic performance of ElastPQ for liver-fibrosis staging was evaluated using the receiver operating characteristic (ROC) curve analysis. To compare the diagnostic accuracy of the ElastPQ, TE, and APRI values, the area under the ROC curve (AUROC) using the normal z-score test was used [32]. The respective cut-off values were determined using a common optimization step that maximized the Youden index [33]. A *p*-value of less than 0.05 was considered statistically significant.

## 3. Results

### 3.1. Patient Characteristics

**Figure 2:**
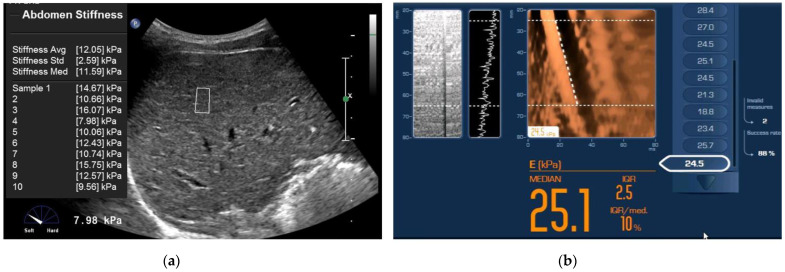
A 48-year-old man with stage F4 fibrosis and an aspartate aminotransferase-to-platelet ratio index of 1.24. Liver stiffness measurement (LSM) using ElastPQ (**a**). The value measured using ElastPQ is shown at the bottom left. The median value is 11.59 kPa. On the transient elastography image, the median LSM was 25.1 kPa (**b**).

The patient characteristics are summarized in Table 1. In this study, 94 patients (77%) had chronic hepatitis B and 20 patients (16%) had chronic hepatitis C. The mean liver stiffness values of ElastPQ (6.1 ± 2.75 kPa) and TE (12.6 ± 9.34 kPa) were significantly different (*p* < 0.001). The mean value of APRI was 1.16 ± 1.7. Specimens with histologically confirmed liver fibrosis were obtained from percutaneous liver biopsy (*n* = 4) and intraoperative liver biopsy (*n* = 12). Ninety-three patients underwent hepatectomy for the following indications: hepatocellular carcinoma (*n* = 82); cholangiocarcinoma (*n* = 3); and combined hepatocellular cholangiocarcinoma (*n* = 8). Five patients underwent a wedge resection for the following indications: metastatic liver cancer (*n* = 3); abscess (*n* = 1); and hemangioma (*n* = 1). The mass was located in the right liver (*n* = 67) or left liver (*n* = 31) and ranged from 1.2 to 7.8 cm in size. The patients were classified as follows: F0 including liver donor (*n* = 10, 8.2%), F1 (*n* = 3, 2.5%), F2 (*n* = 15, 12.3%), F3 (*n* = 37, 30.3%), and F4 (*n* = 57, 46.7%). Representative cases from our population are illustrated in Figure 2.

### 3.2. Relationship between Histologic Fibrosis Stages and the Values on ElastPQ, TE, and APRI

**Figure 3:**
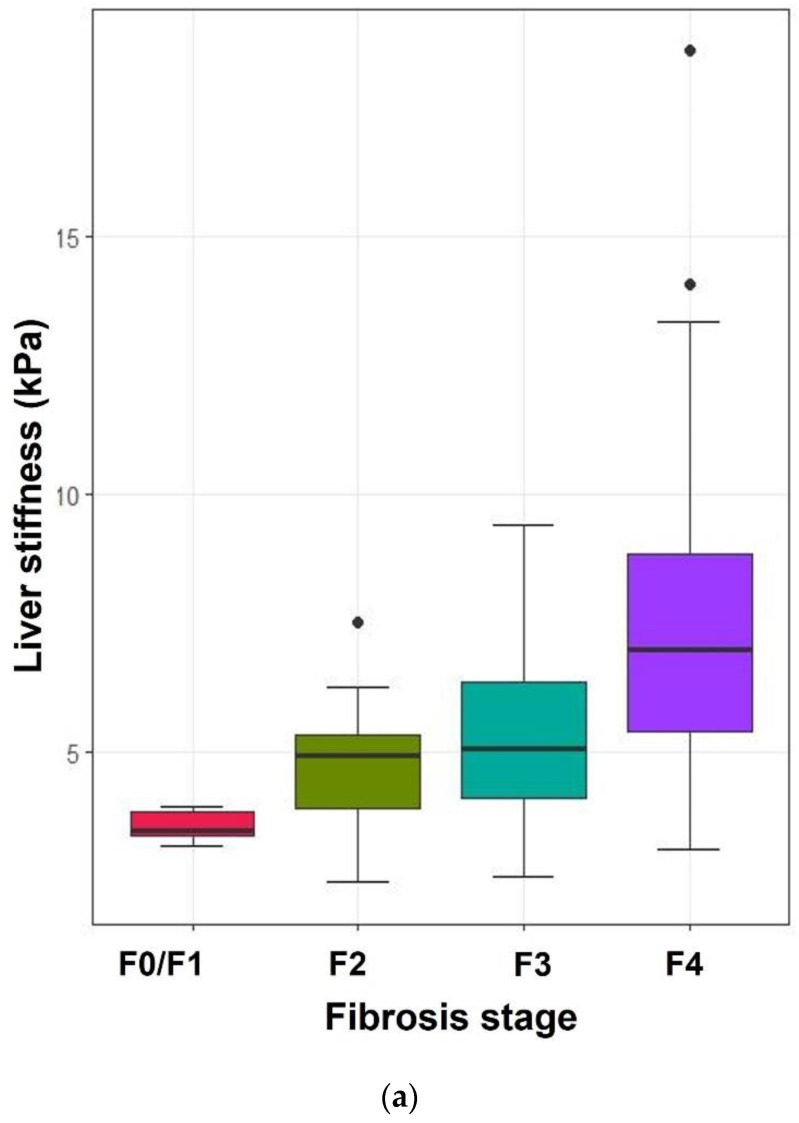

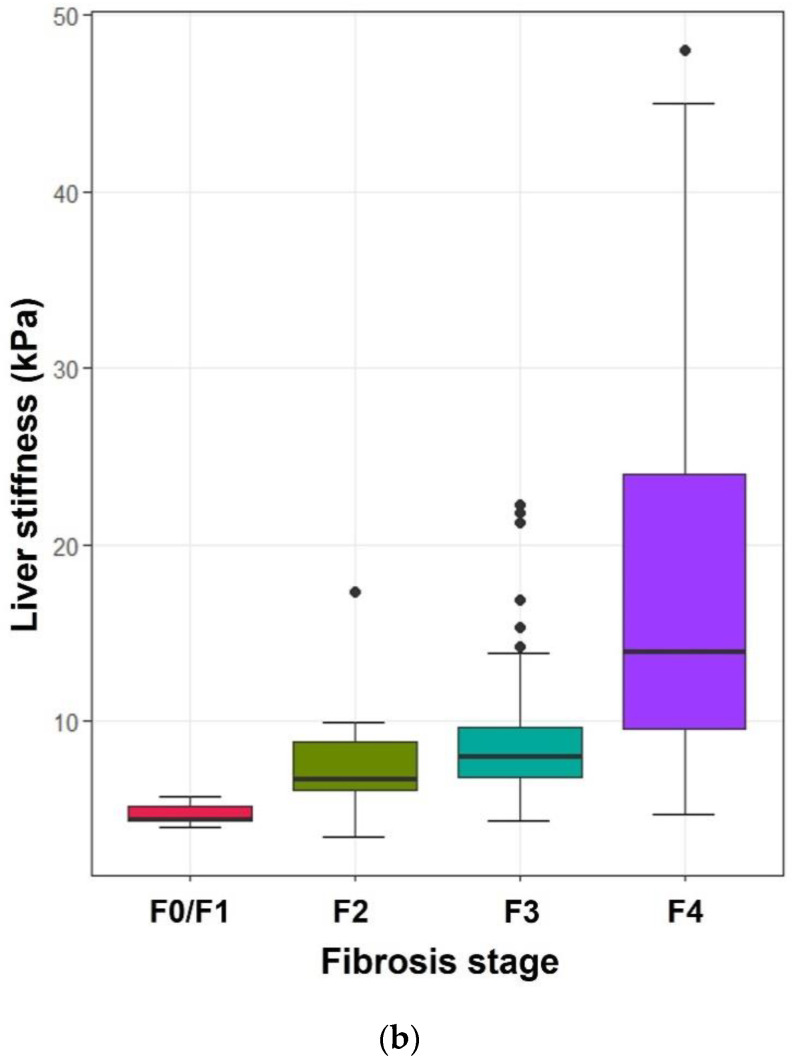
Box-and-whisker plot of ElastPQ and TE at each fibrosis stage. The boxes represent the interquartile range and the thick lines within the boxes represent the median values measured using ElastPQ (**a**) and TE (**b**). The error bars indicate the smallest and largest values within 1.5 box lengths of the 25th and 75th percentiles, respectively. The dots are outliers representing very large values that significantly deviate from the range of observed data.

The mean liver stiffness values measured by ElastPQ and TE at each fibrosis stage are shown in Table 2 and Figure 3. The mean liver stiffness values of each fibrosis stage were significantly different (*p* < 0.05), except between F2 and F3 (*p* = 0.15). ElastPQ and TE measurements steadily increased with an increasing degree of fibrosis and were moderately correlated (ρ = 0.694, *p* < 0.01). A statistically significant positive correlation was demonstrated between the liver stiffness values on ElastPQ, TE, and APRI and the fibrosis stages (ρ = 0.556, ρ = 0.657, ρ = 0.375, respectively; *p* < 0.001).

### 3.3. Diagnostic Accuracy and Cut-Off Values of ElastPQ

Table 3 shows the diagnostic accuracy of ElastPQ in diagnosing the liver fibrosis stage. For significant fibrosis (≥F2), the AUROC value was 0.917 (95% confidence interval [CI], 0.867–0.967) with 83.49% sensitivity, 100% specificity, and a cut-off value of 3.935. For cirrhosis (=F4), the AUROC value was 0.777 (95% CI, 0.693–0.861) with 80.7% sensitivity, 67.69% specificity, and a cut-off value of 5.17.

### 3.4. Comparison of the Diagnostic Performance of ElastPQ, TE, and APRI

**Figure 4:**
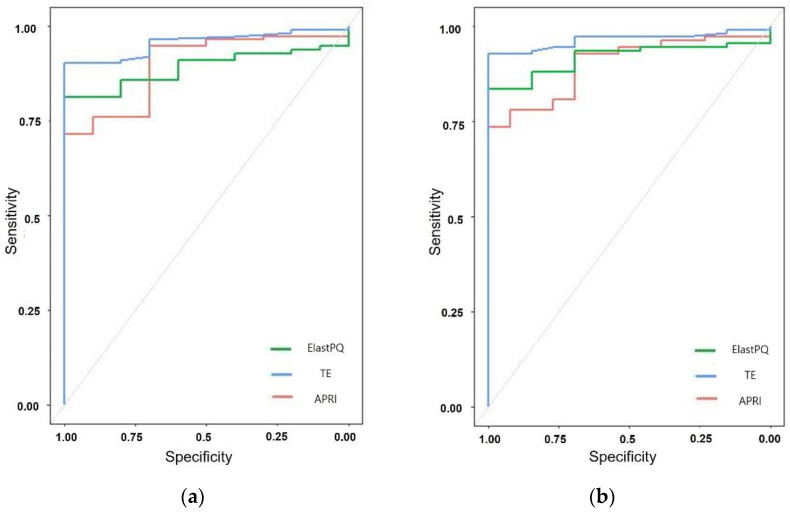

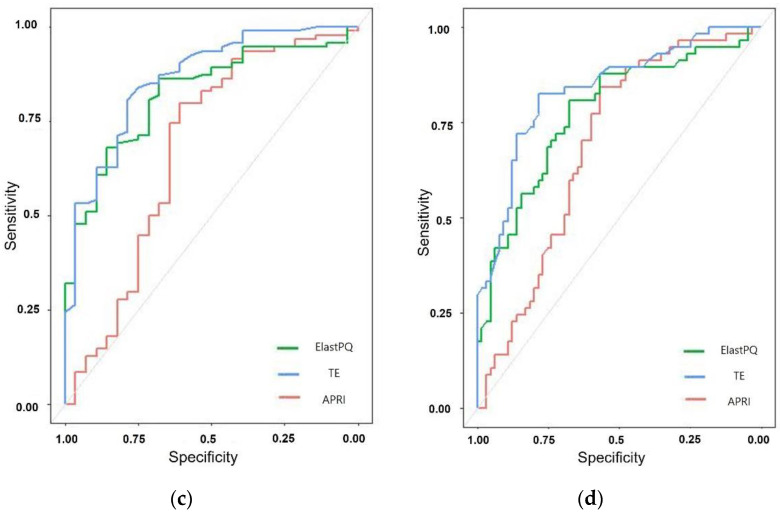
The receiver operating characteristic curves of ElastPQ, transient elastography (TE), and aspartate aminotransferase-to-platelet ratio index (APRI) for predicting fibrosis stage ≥F1 (**a**), ≥F2 (**b**), ≥F3 (**c**), F4 (**d**).

The comparison of the diagnostic performance of ElastPQ, TE, and APRI with the histopathologic grade is summarized in Table 4 and Figure 4. The AUROC values of ElastPQ for the diagnosis of each stage of fibrosis were higher than those of APRI and lower than those of TE. However, these values were not significantly different (*p* > 0.05). For the diagnosis of significant fibrosis (≥F2), the AUROC values of ElastPQ, TE, and APRI were 0.917, 0.964, and 0.896, respectively. For the diagnosis of cirrhosis (=F4), the AUROC values of ElastPQ, TE, and APRI were 0.777, 0.836, and 0.689, respectively.

## 4. Discussion

In this study, we compared the diagnostic performance of ElastPQ, TE, and APRI for liver fibrosis staging, based on the histologic stage of liver fibrosis as a reference standard. ElastPQ showed good diagnostic performance which was higher than that of APRI and similar to that of TE; the AUROC values of ElastPQ, TE, and APRI were 0.917, 0.964, and 0.896, respectively, for the diagnosis of significant fibrosis and 0.777, 0.836, and 0.689, respectively, for predicting F4. Fouad et al. [34] reported that the AUROC values of ElastPQ, TE, and APRI were 0.75, 0.95, and 0.77, respectively, for non-advanced fibrosis, and 0.83, 0.99, and 0.77, respectively, for advanced fibrosis, which is similar to our results [34]. Gerber et al. [35] reported that the AUROC values of ElastPQ were comparable to those of TE for predicting the liver fibrosis stage (ElastPQ/TE: F ≥ 2, 0.87/0.92; F = 4, 0.88/0.9) in patients with chronic liver disease.

The diagnostic performance of TE was better than that of ElastPQ. However, this difference was not statistically significant (*p* > 0.05). This suggests that both ElastPQ and TE can be used to evaluate liver fibrosis. However, ElastPQ has several advantages over TE. ElastPQ, which is implemented in conventional real-time US systems, can be performed as part of a standard liver US examination and could help select the region of interest without blood vessels, bile ducts, or rib shadows [36].

Measurements of ElastPQ showed a significant positive correlation with the histologic fibrosis stage (ρ = 0.556, *p* < 0.001). In agreement with our results, Fouad et al. [34] reported a significant correlation between the liver-stiffness measurements obtained by ElastPQ and the different stages of histologic liver fibrosis (ρ = 0.68, *p* < 0.0001). Bucsics et al. [1] reported that ElastPQ measurements steadily increased with an increasing degree of liver fibrosis (ρ = 0.7025, *p* < 0.001). However, in the previous study, the liver fibrosis stage was determined by TE. A formidable advantage of our study is that we used the histopathologic liver fibrosis stage as the reference standard. In comparison with other studies, the correlation coefficient was low because liver stiffness values were also affected by other factors, such as inflammation and the skin-to-liver distance [37,38].

Our results showed that ElastPQ demonstrated good diagnostic performance in determining the liver fibrosis stage in patients with chronic viral hepatitis. These results are in good accordance with the results of other studies of patients with chronic viral hepatitis, non-alcoholic fatty liver disease, chronic liver disease, and autoimmune liver disease [19,20,22,34]. Among the AUROCs for identifying each stage of liver fibrosis, ElastPQ showed good performance in determining F2 with an AUROC value of 0.917 with high sensitivity (83.49%) and specificity (100%). This result suggests that ElastPQ can be used as an optimal screening tool for the diagnosis of significant fibrosis. Thus, if ElastPQ can optimally determine the F2 stage, it may be possible to reduce the number of patients who progress to liver cirrhosis [39]. However, our study showed a relatively lower diagnostic performance with an AUROC value of 0.777 for predicting liver cirrhosis, unlike in a previous study [34]. Compared with other studies, our study included a relatively large number of patients with cirrhosis, which could lead to the variability of liver stiffness measurements and overlap with other fibrosis stages.

The best cut-off values for predicting significant fibrosis, advanced fibrosis, and cirrhosis were 3.935, 3.97 and 5.17 kPa, respectively, which were lower than the values reported in previous studies [20,24,34]. Cut-off values of ElastPQ are often affected by the type of US equipment used and the etiology of the underlying chronic liver disease [40]. Our study had an unequal distribution of cases with different fibrosis stages which could have resulted in the variation of shear wave velocity measurements [9]. In the previous study, most patients had chronic viral hepatitis caused by HCV, unlike in our study [34]. Chronic hepatitis B has the tendency of causing macronodular, heterogeneous parenchymal changes, and the total amount of fibrosis could be lower than that observed in chronic hepatitis C [26]. In the absence of the acute exacerbation of inflammation in the HBV group, the steady low-grade inflammation in HCV is considered to be associated with higher ElastPQ values. Indeed, the cut-off values in previous studies were variable, which could be owing to the various underlying liver diseases.

Our study has several limitations. First, as mentioned before, the different stages of fibrosis were not uniformly balanced in our series. In particular, the very small number of patients with fibrosis stage F1 (*n* = 3) compared to those of stages F3–F4 may have affected some statistical analyses. We included a relatively small number of patients in the chronic hepatitis C group compared to patients in the chronic hepatitis B group. These distributions could have influenced the optimal cut-off values. Second, the analysis was performed in a relatively small number of patients at a single study center. Thus, it would be necessary to validate these results in larger multicenter studies.

## 5. Conclusions

ElastPQ is a promising noninvasive method for the evaluation of liver fibrosis in chronic viral hepatitis with a good diagnostic performance comparable to that of TE. Furthermore, compared to TE, ElastPQ has the advantage of imaging liver stiffness in real time while guided by a B-mode image.

## Figures and Tables

**Table 1:** Patient demographics and clinical features.

Characteristic	Value (*n* = 122)
Patient age (y)	57.3 ± 11.6 (18–76)
Sex	
Male	93 (76)
Female	29 (24)
Body mass index (kg/m^2^)	23.7 ± 3.1 (15.8–34)
ElastPQ (kPa)	6.1 ± 2.75 (2.5–18.6)
Transient elastography (kPa)	12.6 ± 9.34 (3.5–48)
Blood marker	
AST (IU/L)	56.8 ± 68.7 (11–513)
ALT (IU/L)	45.98 ± 53.28 (6–348)
ALP (IU/L)	88.4 ± 44.6 (27–364)
Albumin (g/dL)	3.8 ± 0.5 (0.8–4.9)
GGT(IU/L)	85.2 ± 108.2 (11–654)
Platelet count (10^3^/mm^3^)	153 ± 61 (11.5–339)
APRI	1.16 ± 1.7 (0.13–14.41)
Underlying liver disease	
Chronic hepatitis B	94 (77)
Chronic hepatitis C	20 (16)
Fibrosis stage (Metavir score)	
F0 *	10 (8.2)
F1	3 (2.5)
F2	15 (12.3)
F3	37 (30.3)
F4	57 (46.7)

Note—* F0 included living liver donor. Data are expressed as means ± standard deviations (range) and numbers (%). AST: aspartate aminotransferase; ALT: alanine aminotransferase; ALP: alkaline phosphatase; GGT: gamma-glutamyl transferase; APRI: AST-to-platelet ratio index.

**Table 2:** Values of ElastPQ, TE, and APRI according to the fibrosis stage.

Histologic Fibrosis Stage	ElastPQ	TE	APRI
F0/1	3.51 ± 0.14 (3.18–3.94)	4.71 ± 0.57 (4.0–5.7)	0.28 ± 0.10 (0.18–0.46)
F2	4.74 ± 1.27 (4.03–5.45)	7.63 ± 3.24 (3.5–17.3)	1.15 ± 0.94 (0.14–2.96)
F3	5.48 ± 1.76 (2.58–9.39)	9.47 ± 4.65 (4.4–22.3)	0.96 ± 1.36 (0.13–7.83)
F4	7.52 ± 3.09 (3.1–18.6)	17.71 ± 10.86 (4.7–48.0)	1.29 ± 1.29 (0.17–5.36)

Note—mean ± standard deviation (95% CIs). All data are given in kilopascals.

**Table 3:** Diagnostic performance of ElastPQ.

Parameter	Stage ≥ F1	Stage ≥ F2	Stage ≥ F3	Stage F4
AUROC	0.890 (0.832–0.949)	0.917 (0.867–0.967)	0.822 (0.742–0.902)	0.777 (0.693–0.861)
Criterion (kPa)	3.935	3.935	3.97	5.17
Sensitivity (%)	81.25	83.49	86.17	80.7
Specificity (%)	100	100	67.86	67.69

Note—numbers in parentheses are 95% CIs. AUROC: the area under the receiver operating characteristics curve.

**Table 4:** AUROC for the diagnostic accuracies of ElastPQ, TE, and APRI scoring in patients with different Metavir fibrosis stage.

Fibrosis Assessment Method	AUROC	Cut-Off Value (kPa)	Sensitivity (%)	Specificity (%)	*p* Value
Stage ≥ F2					
ElastPQ	0.917 (0.867–0.967)	3.935	83.49	100	<0.001
TE	0.964 (0.914–0.990)	5.7	92.66	100	<0.001
APRI	0.896 (0.828–0.944)	0.46	71.56	100	<0.001
Stage = F4					
ElastPQ	0.777 (0.693–0.861)	5.17	80.70	67.69	<0.001
TE	0.836 (0.764–0.908)	8.9	82.46	78.46	<0.001
APRI	0.689 (0.594–0.784)	0.47	84.21	56.92	<0.001

Note—AUROC: the area under the receiver operating characteristics curve. There was no statistical difference in the AUROC between each test.
